# An Important Biomarker in Patients with Bell’s Palsy: Serum Calprotectin

**DOI:** 10.3390/medicina61040747

**Published:** 2025-04-18

**Authors:** Cihan Türker, Elif Emre, Süleyman Aydın, Mustafa Dalgıç, Deniz Baklacı

**Affiliations:** 1 Department of Otolaryngology, Private Medical Hospital, Elazig 23040, Turkey; cihan_turker23@hotmail.com; 2 Department of Anatomy, Firat University Faculty of Medicine, Elazig 23200, Turkey; elifkaplan1.1@gmail.com; 3 Department of Biochemistry, Firat University Faculty of Medicine, Elazig 23200, Turkey; saydin1@firat.edu.tr; 4 Department of Otolaryngology, Ataturk State Hospital, Zonguldak 67030, Turkey; 5 Department of Otolaryngology, Bulent Ecevit University Faculty of Medicine, Zonguldak 67000, Turkey; doktorent@gmail.com

**Keywords:** Bell’s palsy, calprotectin, marker

## Abstract

*Background and Objectives*: This study aimed to examine the relationship between serum calprotectin levels and facial paralysis in patients with Bell’s palsy and to determine its prognostic significance. *Materials and Methods*: This study included 40 patients diagnosed with Bell’s palsy and 20 healthy individuals as controls. The patients were categorized into three groups based on their response to treatment: complete response, partial response, and no response. Blood samples were taken before treatment and in the third month after treatment to measure C-reactive protein, white blood cell count, lymphocyte count, neutrophil count, neutrophil-to-lymphocyte ratio, and calprotectin levels. *Results*: Serum calprotectin levels were found to be elevated in patients with BP compared to the healthy controls; however, no significant correlation was observed between calprotectin levels and disease prognosis. *Conclusions*: The findings suggest that Bell’s palsy patients have elevated serum calprotectin levels compared to healthy individuals, indicating the potential use of calprotectin as a biomarker in Bell’s palsy. However, no significant difference in calprotectin levels was observed between patients with varying degrees of treatment response, suggesting that calprotectin may be limited in predicting disease prognosis.

## 1. Introduction

Acute peripheral facial paralysis (APFP) is characterized by acute-onset facial nerve paralysis, typically affecting one side of the face. Idiopathic peripheral facial paralysis, commonly known as Bell’s palsy (BP), accounts for approximately 70% of APFP cases and occurs with similar frequency among men and women [1]. Although the exact mechanism of BP remains unclear, it is thought to be related to the reactivation of herpes simplex virus type 1 or varicella-zoster virus. In addition to viral factors, tumors, trauma, anatomical anomalies, inflammation, ischemia, and cold exposure have also been implicated as etiological factors [2].

The primary treatment of BP involves corticosteroids and antiviral medications, although the response to therapy varies. While the majority of patients experience full recovery within three to six months, there are cases where no recovery occurs [3]. Therefore, determining the prognosis of the disease is of critical importance.

The severity of facial paralysis is a key factor in predicting prognosis. The House–Brackmann and Sunnybrook grading systems are commonly used to assess the degree of paralysis. Electrophysiological modalities such as electroneurography, electromyography, and nerve stimulation tests are also employed to evaluate prognosis. Electroneurography is highly correlated with disease prognosis, providing an indication of facial nerve degeneration. It is particularly important for patients scheduled for facial nerve decompression. Electromyography and nerve stimulation tests are auxiliary tools in predicting prognosis; however, these tests are typically conducted at least three days after symptom onset [4]. Thus, there is a need for additional biomarkers to predict facial paralysis prognosis earlier. To this end, various inflammatory, metabolic, and hematological markers have been investigated [5,6].

Inflammatory causes have been implicated in the etiology of BP. According to the inflammation theory, the facial nerve becomes inflamed, causing swelling of the nerve sheath, which can lead to entrapment in the labyrinthine segment, resulting in paralysis [7]. Several studies have thus examined various inflammatory markers and immune system cells in patients with BP. Among the most frequently examined parameters are the neutrophil-to-lymphocyte ratio (NLR), C-reactive protein (CRP), albumin, and procalcitonin.

Calprotectin is a major antimicrobial protein belonging to the S100 family of proteins. It enters the systemic circulation during inflammation through neutrophil activation or the adhesion of monocytes to the epithelium, and is therefore considered an inflammatory biomarker [8].

This study aimed to explore the relationship between serum calprotectin levels and facial paralysis in patients diagnosed with BP and to determine its prognostic significance.

## 2. Materials and Methods

### 2.1. Study Design

A retrospective study was carried out, between 1 January 2023 and 30 June 2024, within a tertiary referral center hospital. This study included 40 patients diagnosed with BP, as well as a control group of 20 healthy individuals. A complete ear–nose–throat and head and neck examinations were performed for all patients. Complete blood count (CBC), serum calprotectin, pure tone audiometry, stapes reflex, routine blood biochemistry, and viral serological markers were obtained from the patients diagnosed with unilateral peripheral facial paralysis in order to exclude any other causes of facial paralysis. Additionally, contrast-enhanced MRI and temporal CT were obtained from all patients at the onset of the treatment. The diagnosis of Bell’s palsy was confirmed by at least two otolaryngology specialists in the patient group. In the control group, the CBC was obtained and no other tests were performed.

### 2.2. Inclusion and Exclusion Criteria

This study included 40 patients diagnosed with BP who were treated at a tertiary referral center hospital, as well as a control group of 20 healthy individuals. All patients underwent full head and neck examination, and other causes of facial palsy were excluded. This study included patients with unilateral peripheral facial palsy that developed rapidly (within 72 h) and had no other identifiable causes. Patients with any autoimmune disease, rheumatologic disease, acute or chronic infection, cardiovascular disease, diabetes mellitus, metabolic syndrome, chronic obstructive pulmonary disease, amyloidosis, chronic renal failure, obstructive sleep apnea, active smoking, or Otological diseases were excluded from this study. The control group consisted of healthy volunteers without ear pathology or systemic disorder and with normal audiological findings.

### 2.3. Study Setting and Population

The patient group consisted of 20 males and 20 females, while the control group included 10 males and 10 females. The mean age was 44.17 ± 18.33 years in the BP group and 44.50 ± 10.72 years in the control group. The patients were administered a standard treatment protocol consisting of steroids and antiviral drugs. The patients in the BP group were further divided into three subgroups based on their response to treatment: complete response, partial response, and no response. Blood samples were taken from the patients before treatment and at the third month of follow-up to measure CRP, white blood cell (WBC) count, lymphocyte count, neutrophil count, NLR, and calprotectin levels. These values were compared within the BP group and with the values obtained from the healthy control group. The effect of inflammatory markers on patient prognosis was also investigated.

### 2.4. Blood Sampling

All blood samples were drawn by same personnel and immediately analyzed without any delay. The Beckman Coulter LH 750 automated complete blood analyzer device (Beckman Coulter, Pasadena, CA, USA) was used to analyze complete blood count (CBC) parameters. Calprotectin was analyzed with a ChroMate, Microplate Reader P4300 devices (Awareness Technology Instruments, Palm City, FL, USA), and the results were reported in ng/mL.

### 2.5. Methods of Data Collection

Data were collected between 1 January 2023 and 30 June 2024. All data were collected and organized in a structured database using Microsoft Excel. Data entry was performed by trained personnel. Our database was regularly checked for any missing or inconsistent data. Data analyses were conducted based on the data stored in this Excel database.

### 2.6. Statistical Analysis

Descriptive statistics for continuous data were presented as mean ± standard deviation, median, minimum, and maximum values, while categorical data were presented as numbers and percentages. The Shapiro–Wilk test was used to assess the normality of the distribution of continuous variables. The Mann–Whitney U test was employed to compare continuous data between two groups. The Wilcoxon test was used to compare pre- and post-treatment measurements within the patient group. The Chi-square test was applied for group comparisons of nominal variables (in cross-tables). IBM SPSS version 20 (Chicago, IL, USA) was used for statistical analyses, with statistical significance set at *p* < 0.05.

## 3. Results

The mean age of the BP group was 44.17 ± 18.33 years, similar to the control group’s mean of 44.50 ± 10.72 years (*p* = 0.815). No significant difference was observed in gender distribution between the two groups (*p* > 0.05) (Table 1). Except for Bell’s palsy, the patients were otherwise healthy.

**Figure 1:**
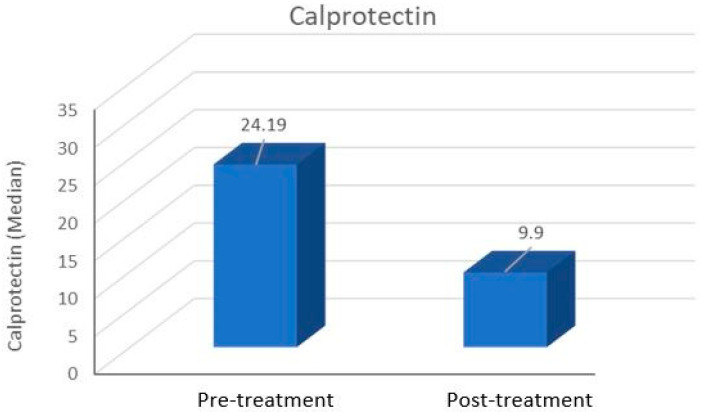
Pre- and post-treatment calprotectin values of patients with Bell’s palsy.

CRP levels significantly decreased after treatment compared to pre-treatment values (*p* < 0.001), as did WBC counts (*p* < 0.001). However, there was no significant change in lymphocyte levels (*p* > 0.05). Neutrophil counts and NLR were also significantly lower after treatment (*p* < 0.01 and *p* < 0.05, respectively) (Table 2). Similarly, calprotectin levels showed a significant reduction after treatment (*p* < 0.001) (Figure 1).

When comparing pre-treatment values between the BP and control groups, WBC counts (*p* < 0.001), neutrophil counts (*p* < 0.001), NLR (*p* < 0.01), and calprotectin levels (*p* < 0.05) were significantly higher in the BP group, while CRP and lymphocyte values did not differ significantly (*p* > 0.05) (Table 3).

Post-treatment comparisons revealed that the BP group had lower CRP levels than the control group (*p* < 0.05) and higher neutrophil counts (*p* < 0.01) and NLR (*p* < 0.05). No significant differences were observed in post-treatment WBC or lymphocyte counts or calprotectin levels between the two groups (*p* > 0.05).

Following treatment, 18 patients (45%) in the BP group achieved full recovery, 15 (37.5%) showed partial recovery, and 7 (17.5%) did not respond to treatment. The calprotectin levels of the BP group did not significantly differ between patients with complete response, partial response, or no response, either before or after treatment (*p* > 0.05 for both) (Table 4).

## 4. Discussion

Inflammatory causes are considered significant in the pathogenesis of BP [7]. Accordingly, several studies have examined the relationship between BP and immune system cells and biomarkers, focusing primarily on lymphocytes, neutrophils, monocytes, and cellular immune responses [9]. Research indicates elevated levels of inflammatory markers, such as interleukin-1 (IL-1), IL-6, IL-8, and tumor necrosis factor-α, in patients with BP [10,11]. Furthermore, NLR has been shown to increase significantly in patients with BP, and its relevance in predicting disease prognosis has been emphasized. Studies have also reported that NLR is higher in patients with BP who have not fully recovered compared to those who have achieved complete recovery, suggesting that NLR can be used to predict prognosis in BP [12,13]. Supporting these findings, the current study revealed a significant decrease in NLR after treatment compared to pre-treatment values (*p* < 0.05). Additionally, post-treatment NLR values in the BP group were significantly higher than those in the control group (*p* < 0.05).

In a study by Kim et al., patients who did not respond fully to treatment had lower WBC and eosinophil counts after seven days of therapy than those with a complete treatment response. Similarly, lower lymphocyte counts were observed in non-responders before and after seven days of treatment compared to complete responders [14]. In contrast, our study found no statistically significant difference in lymphocyte levels before and after treatment (*p* > 0.05). Furthermore, no significant difference was observed between post-treatment lymphocyte levels between the BP and control groups (*p* > 0.05).

In recent years, studies exploring the relationship between serum calprotectin levels and otolaryngological diseases have increased in number. One study found a correlation between symptom severity and serum calprotectin levels in patients with chronic rhinosinusitis [15]. In another study, Kuzucu et al. demonstrated higher serum calprotectin levels in patients diagnosed with idiopathic sudden sensorineural hearing loss compared to healthy individuals. The authors also reported higher calprotectin levels in patients who did not respond to treatment compared to those with partial or full recovery, suggesting that serum calprotectin may be a marker of disease severity and treatment response in idiopathic sudden sensorineural hearing loss [16].

In a study by Van Crombruggen et al., patients with chronic rhinosinusitis and nasal polyposis exhibited higher serum calprotectin levels than the healthy population. The authors suggested that this finding could be attributed to the release of calprotectin from cells due to inflammation [17]. Similarly, Erdoğan et al. reported higher serum calprotectin levels in patients with nasal polyposis compared to healthy controls. They suggested that elevated serum calprotectin served as a robust marker of nasal inflammation and could predict a greater need for nasal and systemic steroid therapy, independent of physical examination or computed tomography findings [18]. Kum et al. demonstrated that patients with obstructive sleep apnea had higher serum calprotectin levels than healthy individuals, and these levels also correlated with the severity of obstructive sleep apnea [19].

In this study, pre-treatment levels of CRP, WBC, neutrophils, NLR, and serum calprotectin were significantly higher in patients with BP compared to their post-treatment values (*p* < 0.05). No significant difference was found in lymphocyte levels before and after treatment (*p* > 0.05). Additionally, pre-treatment levels of CRP, WBC, neutrophils, NLR, and serum calprotectin were higher in patients with BP compared to the healthy population (*p* < 0.05), while lymphocyte levels showed no significant difference (*p* > 0.05). Furthermore, no significant difference was observed in calprotectin levels between patients who achieved full recovery, partial recovery, or no response, either before or after treatment (*p* > 0.05).

Our study is the first in the literature to investigate the relationship between BP and serum calprotectin levels. The findings suggest that BP patients have elevated serum calprotectin levels compared to healthy individuals, indicating the potential use of calprotectin as a biomarker in BP. However, no significant difference in calprotectin levels was observed between patients with varying degrees of treatment response, suggesting that calprotectin may be limited in predicting disease prognosis.

## 5. Conclusions

This study showed elevated serum calprotectin levels in patients with BP compared to the healthy population. However, no significant correlation was found between calprotectin levels and disease prognosis.

## Figures and Tables

**Table 1:** Comparison of age and gender between the BP and control groups.

	BP Group (*n* = 40)	Control Group (*n* = 20)	*p* Value
Age (year), mean ± SD (Min–Max)	44.17 ± 18.33 (14–74)	44.50 ± 10.72 (15–64)	0.815 ^a^
Gender, n (%)			
Male	20 (50)	10 (50)	4.72 ^b^
Female	20 (50)	10 (50)	

^a^ Mann–Whitney U test, ^b^ Chi-square test, BP: Bell’s palsy, SD: standard deviation.

**Table 2:** Comparison of pre- and post-treatment measurements in the BP group.

	Pre-Treatment	Post-Treatment	*p* *
	Mean ± SD Median (Min–Max)	Mean ± SD Median (Min–Max)	
CRP	3.00 ± 2.77 2.5 (0.2–11)	1.36 ± 1.36 1.05 (0–6.4)	<0.001
WBC count	10.94 ± 4.15 10.42 (6.49–20.94)	7.75 ± 1.62 7.40 (5.24–11.33)	<0.001
Lymphocyte count	2.17 ± 1.05 2.19 (0.67–4.37)	1.91 ± 0.58 1.90 (1.03–2.87)	0.519
Neutrophil count	8.16 ± 4.31 7.48 (3.30–18.94)	5.34 ± 1.77 5.08 (2.16–9.04)	0.001
Neutrophil-to-lymphocyte ratio	5.29 ± 4.18 4.17 (1.10–13.31)	3.20 ± 1.89 2.91 (0.86–8.29)	0.038
Calprotectin	29.94 ± 21.49 24.19 (5.61–73.46)	14.45 ± 9.91 9.90 (2.80–35.32)	<0.001
Stage	3 (2–4)	2 (1–3)	<0.001

* Wilcoxon test, BP: Bell’s palsy, SD: standard deviation, CRP: C-reactive protein, WBC: white blood cell.

**Table 3:** Comparison of pre-treatment laboratory parameters between the BP and control groups.

	BP Group (n = 40)	Control (n = 20)	*p* *
Pre-Treatment Parameter	Mean ± SD Median (Min–Max)	Mean ± SD Median (Min–Max)	
CRP	3.00 ± 2.77 2.5 (0.2–11)	2.05 ± 1.89 1.95 (0.2–9.5)	0.322
WBC count	10.94 ± 4.15 10.42 (6.49–20.94)	7.01 ± 1.43 7 (4.80–9.34)	<0.001
Lymphocyte count	2.17 ± 1.05 2.19 (0.67–4.37)	2.17 ± 0.68 2.01 (1.49–3.64)	0.975
Neutrophil count	8.16 ± 4.31 7.48 (3.30–18.94)	3.92 ± 1.42 3.52 (2.37–6.72)	<0.001
Neutrophil-to-lymphocyte ratio	5.29 ± 4.18 4.17 (1.10–13.31)	2.03 ± 0.93 1.93 (0.81–3.65)	0.007
Calprotectin	29.94 ± 21.49 24.19 (5.61–73.46)	14.35 ± 7.72 14.69 (2.19–29.40)	0.012

* Mann–Whitney U test, BP: Bell’s palsy, SD: standard deviation, CRP: C-reactive protein, WBC: white blood cell.

**Table 4:** Comparison of pre- and post-treatment calprotectin levels according to treatment response in the BP group.

	Complete Response (n = 18)	Partial/No Response (n = 22)	*p* *
	Mean ± SD Median (Min–Max)	Mean ± SD Median (Min–Max)	
Pre-treatment calprotectin	33.46 ± 23.95 27.94 (7.48–73.46)	27.06 ± 19.34 22.06 (5.61–66.02)	0.381
Post-treatment calprotectin	12.21 ± 10.91 8.77 (2.8–35.32)	16.29 ± 8.85 15.23 (4.44–30.02)	0.089

* Mann–Whitney U test, BP: Bell’s palsy, SD: standard deviation.

## Data Availability

The original contributions presented in this study are included in the article. Further inquiries can be directed to the corresponding author.
